# Genome-wide DNA methylation profiling reveals novel epigenetic signatures in squamous cell lung cancer

**DOI:** 10.1186/s12864-017-4223-3

**Published:** 2017-11-23

**Authors:** Yuan-Xiang Shi, Ying Wang, Xi Li, Wei Zhang, Hong-Hao Zhou, Ji-Ye Yin, Zhao-Qian Liu

**Affiliations:** 1 Department of Clinical Pharmacology, Xiangya Hospital, Central South University, Changsha, 410008 People’s Republic of China; 2 Institute of Clinical Pharmacology, Central South University, Hunan Key Laboratory of Pharmacogenetics, Changsha, 410078 People’s Republic of China; 3 Hunan Province Cooperation Innovation Center for Molecular Target New Drug Study, Hengyang, 421001 People’s Republic of China

**Keywords:** Lung cancer, DNA methylation, Biomarker, Diagnosis, Epigenetics

## Abstract

**Background:**

Epigenetic alterations are strongly associated with the development of cancer. The aim of this study was to identify epigenetic pattern in squamous cell lung cancer (LUSC) on a genome-wide scale.

**Results:**

Here we performed DNA methylation profiling on 24 LUSC and paired non-tumor lung (NTL) tissues by Illumina Human Methylation 450 K BeadArrays, and identified 5214 differentially methylated probes. By integrating DNA methylation and mRNA expression data, 449 aberrantly methylated genes accompanied with altered expression were identified. Ingenuity Pathway analysis highlighted these genes which were closely related to the carcinogenesis of LUSC, such as ERK family, NFKB signaling pathway, Hedgehog signaling pathway, providing new clues for understanding the molecular mechanisms of LUSC pathogenesis. To verify the results of high-throughput screening, we used 56 paired independent tissues for clinical validation by pyrosequencing. Subsequently, another 343 tumor tissues from the Cancer Genome Atlas (TCGA) database were utilized for further validation. Then, we identified a panel of DNA methylation biomarkers (CLDN1, TP63, TBX5, TCF21, ADHFE1 and HNF1B) in LUSC. Furthermore, we performed receiver operating characteristics (ROC) analysis to assess the performance of biomarkers individually, suggesting that they could be suitable as potential diagnostic biomarkers for LUSC. Moreover, hierarchical clustering analysis of the DNA methylation data identified two tumor subgroups, one of which showed increased DNA methylation.

**Conclusions:**

Collectively, these results suggest that DNA methylation plays critical roles in lung tumorigenesis and may potentially be proposed as a diagnostic biomarker.

**Trial registration:**

ChiCTR-RCC-12002830 Date of registration: 2012–12-17.

**Electronic supplementary material:**

The online version of this article (10.1186/s12864-017-4223-3) contains supplementary material, which is available to authorized users.

## Background

Lung cancer is the leading cause of cancer-related mortality throughout the world [1]. There are two main histological types of lung cancer, non-small cell lung cancer (NSCLC) and small cell lung cancer. NSCLC comprises three major histological subtypes: squamous cell carcinoma (LUSC), adenocarcinoma and large cell carcinoma [2]. Early diagnosis of cancer is one of the most important factors contributing to the successful and effective treatment. However, many patients are diagnosed with advanced lung cancer due to the asymptomatic nature of early stages and lack of effective screening modalities, resulting in a very low five-year survival rates for them. Therefore, it is essential to identify tumor specific molecular biomarkers for risk assessment and effective early screening.

Tumorigenesis involves a multi-step process, which is the result of the interactions of genetic, epigenetic and environmental factors. The change of these factors results in dysregulation of key oncogenes and tumor suppressor genes. Epigenetic mechanisms are heritable and reversible, including DNA methylation, histone modifications and chromatin organization. DNA methylation is a major epigenetic modification which leads to gene silencing at the transcriptional level. It is involved in some crucial biological processes, including proliferation, apoptosis, cell cycle, DNA repair, tumor invasion and metastasis [3]. Thus, identification of DNA methylation biomarkers has emerged as one of the most promising approaches to improve cancer diagnosis, it presents several advantages compared with other markers [4, 5]. Firstly, methylation changes in lung cancer appear to be early events and thus could be used to improve early detection of malignant tumors [6]. Additionally, the DNA methylation represents a very stable sign that can be detected in many different types of samples, including tumor tissues, cancer cells in body fluids [7, 8]. Most importantly, DNA methylation can be detected by a wide range of sensitive and cost efficient techniques even in samples with low tumor purity.

In previous studies, a variety of epigenetic biomarkers has been evaluated in lung cancer for early detection and prognosis prediction, however, most of them focused on a single gene. For example, P16, HOXA11 (Homeobox A11) and SOX17 (SRY-box 17) showed abnormal hypermethylation at their promoters, they were considered as biomarkers for lung cancer detection and prognosis prediction [9–11]. In recent years, many epigenetic biomarkers have been identified by using microarray [12]. However, they have either lacked clinical validation via large sample size or focused on a mix of lung cancer histologies, and thereby limited the ability to identify subtypes. Of note, LUSC and adenocarcinoma shows distinct differences in DNA methylation, expression profiles and lesion location, although they are similarly treated in clinical practice due to the largely unknown underlying molecular mechanisms [13]. Homogeneous treatment strategies have been traditionally implemented for the two fundamentally different subtypes in clinical practice, resulting in poor response to treatment. Therefore, a better understanding of their biological pattern is critical for finding subtype-specific diagnosis and treatment strategies [14]. The aim of this study is to identify epigenetic pattern in LUSC on a genome-wide scale.

## Methods

All data analysis were performed using R (http://www.r-project.org/, version 2.15.0) and Bioconductor [15].

### Patients and tissue collection

The study was approved by the Ethics Committee of Xiangya School of Medicine, Central South University. All the patients provided written informed consents in compliance with the code of ethics of the World Medical Association (Declaration of Helsinki) at the time of surgery for the donation of their tissue for this research. We also obtained the clinical research admission on the Chinese Clinical Trial Registry and the registration number is ChiCTR-RCC-12002830 [16]. All fresh tissues were frozen in liquid nitrogen immediately after resection and stored at −80 °C. Their basic clinical characteristics were summarized in Table 1. In the current study, current smoker and current reformed smoker for ≤15 years were identified as smoker, whereas current reformed smoker for >15 years and never-smoker were defined as non-smoker.

**Table 1:** Clinicopathological characteristics of patients for discovery and clinical validation cohorts

Clinical and pathological variables	Discovery cohort (*N* = 24)	Validation cohort (*N* = 56)
Age (years)		
< 60	13	29
≥ 60	11	27
Gender		
Male	22	54
Female	2	2
Smoking status		
Smoker	19	48
Non-smoker	5	8
Clinical stage		
I-II	14	28
III-IV	10	28
Differentiation		
Well	0	8
Moderate	16	27
Poor	8	21
Lymph node metastasis		
Yes	11	21
No	13	35

### Global methylation analysis

Genome-scale DNA methylation were analyzed by the Illumina Human Methylation 450 K BeadArrays according to manufacturer’s instructions in the laboratory of CapitalBio Corporation (Beijing, China), which quantifies methylation levels (β-value) of 485,577 CpG-sites. Raw fluorescence intensity values were normalized by Illumina Genome Studio software. Normalized intensities were used to calculate β-values, which were calculated from mean methylated (M) and unmethylated (U) signal intensities for each locus of each sample using the formula (β = (M)/(U + M + 100)). All methylation data analysis was carried out by using R software (v2.1.5). First, we performed data quality control as following steps: 14,511 sites containing missing values were removed, 89,808 sites containing SNPs were removed, 10,245 sites on the X or Y chromosome were removed, 14 sites with *P* value greater than 0.05 in at least 75% samples were removed, and finally 371,000 sites were retained from the original 485,577 sites. Secondly, site-level differential methylation analysis was performed: locus-by-locus analyses was conducted using the nonparametric Wilcoxon rank-sum test, and multiple comparisons correction was performed using Benjamini-Hochberg (BH) FDR from the package in R. Probes with FDR *P*-value <0.05 and β difference ≥ 0.2 were used to identify significantly differential DNA methylation, 5214 sites (1771 genes) were differentially methylated (Additional file 1: Figure S1).

### Global gene expression analysis

Genome-scale mRNA expression profiles were detected by the Human 4 × 180 K expression microarray (Agilent Technologies, Santa Clara, California, USA). After strict data preprocessing and quality control, 32,205 sites were retained from the original sites. We analyzed differential expression using paired t-tests and Benjamini-Hochberg (BH) multiple comparisons correction. Corrected *P*-value <0.05 and absolute fold change >2 were used to identify significantly differential expressed mRNAs, and 3635 genes were differentially expressed.

### Select the validation genes

In order to select the target genes, we designed our study into three steps. Firstly, to identify genes with the greatest changes, we further set up a fourfold cutoff to the average change in gene expression, the results show that 44 genes were coordinately hypermethylated and downregulated in tumors, and 26 genes were coordinately hypomethylated and up-regulated (Additional file 2: Table S3). Secondly, we looked at the literature one by one, looking for genes that were involved in the development of lung cancer and were not reported/reported less from the 70 negatively correlated genes. Finally, we selected several genes for clinical validation, and six genes (CLDN1, TP63, TBX5, TCF21, ADHFE1 and HNF1B) were identified.

### Pyrosequencing analysis

Genomic DNA was extracted from samples by using QIAamp DNA Mini Kit (QIAGEN, Hilden, Germany), following the manufacturer’s instructions. The genomic DNA was bisulfite-modified using an EpiTect Bisulfite Kit (QIAGEN, Hilden, Germany), according to the manufacturer’s instruction. Primer design was carried out using the PyroMark Assay Design 2.0 software; one of the primers was biotinylated to enable capture by Streptavidin Sepharose (Additional file 2: Table S1). Bisulfite-treated DNA was amplified, followed by pyrosequencing using the Gold Q96 CDT Reagents (QIAGEN, Hilden, Germany).

### Quantitative reverse transcription-polymerase chain reaction (qRT-PCR)

qRT-PCR was used to examine the mRNA expression as described previously [17]. Total RNA was extracted from samples with Trizol reagent (Takara, Dalian, China) and then reverse transcribed to cDNA using PrimeScriptTM RT-PCR Kit (Takara, Dalian, China). Real-time PCR was performed using SYBR® Premix DimerEraser™ (Perfect Real Time) (Takara, Dalian, China) in Roche LightCycler 480 II Real-Time PCR system (Roche Diagnostics Ltd., Rotkreuz, Switzerland). The data were calculated using the comparative cycle threshold (CT) (2-ΔΔCT) method. All primers were provided in Additional file 2: Table S1. The differences of mRNA expression level were compared by t test using SPSS 18.0 (SPSS Inc., Chicago, Illinois, USA).

### Functional classification, the cancer genome atlas (TCGA) data and receiver operating characteristics (ROC) analysis

Gene Ontology analyses were performed by using the DAVID Functional Annotation Tool [18]. Gene network and pathway analyses were conducted by IPA (http://www.ingenuity.com). The NextBio database (http://www.nextbio.com) was used to analyze the overlap between our bioset and the other three most highly correlated NextBio biosets.

DNA methylation datasets in LUSC were downloaded from the Cancer Genome Atlas (TCGA) data portal (http://tcga-data.nci.nih.gov). We selected 343 tumor and 39 paired NTL samples, with both DNA methylation data and clinical features information available for performing the correlation analysis. Receiver operating curves were used to assess the predictive capacity of each marker. Area under the curve (AUC) was computed for each curve, and 95% confidence intervals (CI) were also estimated by bootstrapping with 1000 iterations.

## Results

### Genome-wide DNA methylation patterns in LUSC

In our study, a total of 24 LUSC and matched adjacent NTL tissues were analyzed, the strategy was diagrammatically outlined in Additional file 1: Figure S1. Single-CpG-site methylation levels are quantified by β, β ranges from zero (the CpG site is unmethylated) to one (the CpG site is fully methylated). Firstly, we investigated the overall distribution of methylation level in tumor versus NTL, the results showed a bimodal distribution of methylation (Additional file 3: Figure S2A). Normally, the methylation site can be grouped based on their positional context relative to closest CpG island (CGI) and the nearby transcripts (Additional file 3: Figure S2B). Thus, we further identified the methylation level distribution of probes located in five CpG island-based regions (CGIs, south and north shores, and south and north shelves) and six gene-based regions (TSS1500, TSS200, 5′-UTR, first exon, gene body, and 3′-UTR). As indicated in Additional file 3: Figure S2C, we found that most CpG sites in CGIs were hypomethylated as showed by a single peak with the β-value *<*0.2, while CpG sites in CGI shelf regions (both north and south) were hypermethylated as showed by a single peak with the β-value *>*0.6. In addition, CpG sites in CGI shore regions had variable methylation levels as indicated by a bimodal distribution, and this pattern is symmetric in the north and south shores of CGIs. In brief, the DNA methylation levels gradually increased with the CpG sites far away from CGIs. We further investigated that methylation patterns at gene context based on genomic content, the first exon and its upstream area (TSS1500, TSS200, 5′-UTR) are hypomethylated, while gene body and 3′-UTR are hypermethylated (Additional file 3: Figure S2D). We found that the CpG sites which closer to 3′-UTR have higher methylation levels. We also compared the methylation level between LUSC and NTL samples based on these groups, although the above distribution curves are similar with each other, our statistical analysis indicated that there were significant differences in LUSC versus NTL tissues (Additional file 2: Table S2).

### Methylation differences in LUSC and matched NTL tissue

**Fig. 1:**
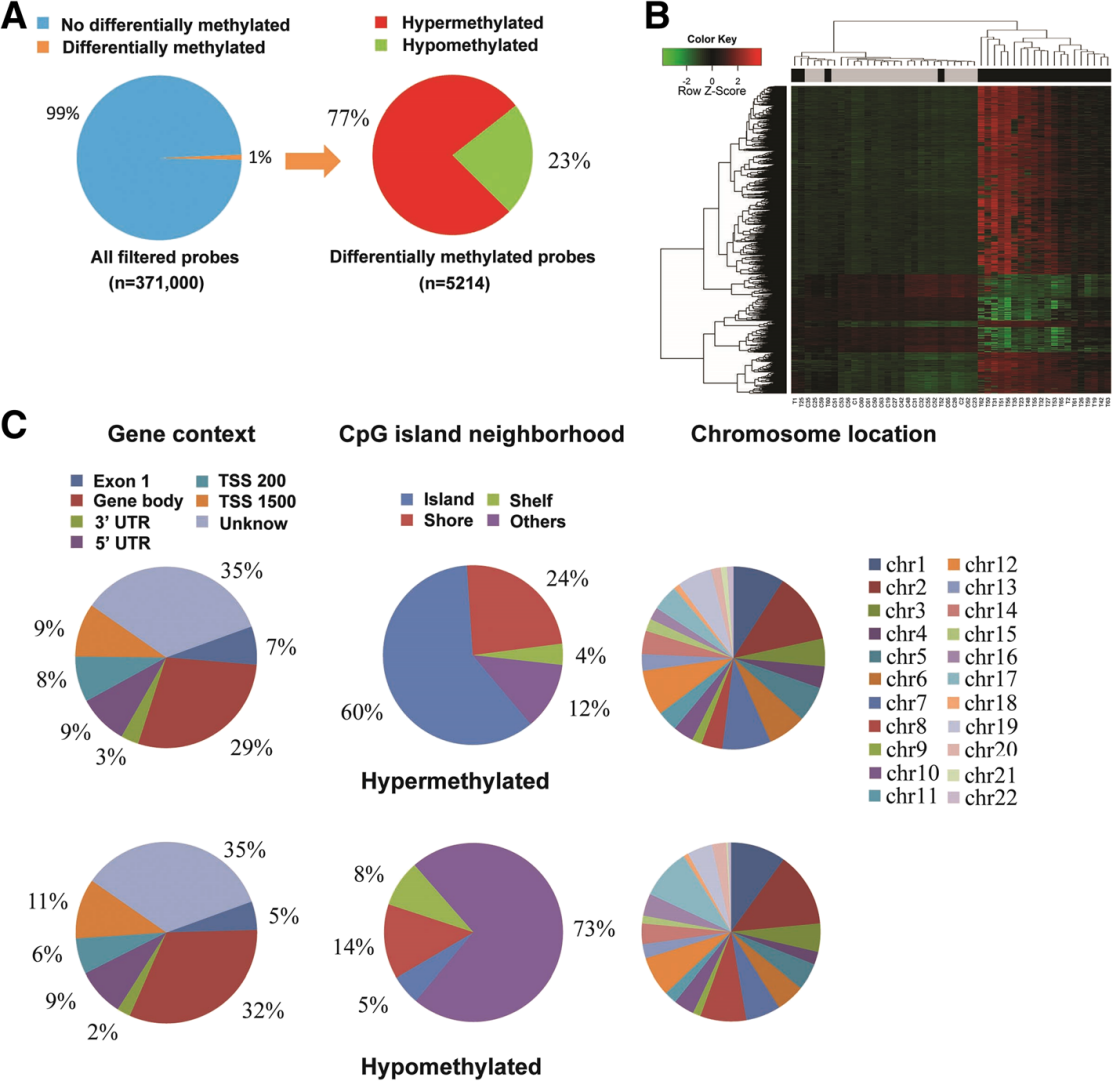
Identification of DNA methylation differences between LUSC and NTL. **a** Pie charts showed the distribution of all filtered probes retained from the microarray, and revealed the methylation differences in LUSC and matched NTL tissues. **b** Two-dimensional hierarchical clustering was performed using the 5214 variable DNA methylation probes across all samples (*n* = 48). **c** The genomic distribution of differentially methylated probes in the gene context, CpG-site neighborhood and chromosome, respectively. TSS: transcription start site, UTR: untranslated region, Chr: chromosome

Then, we analyzed the methylation differences in LUSC and matched NTL tissues. After normalization, 371,000 probes from the methylation array were retained for analysis. Using the criteria of FDR *p*-value <0.05 and β difference ≥ 0.2, we identified 5214 probes (1771 gene) differentially methylated. Among them, 4001 probes (77%) were significantly hypermethylated, and 1213 probes (23%) were significantly hypomethylated in tumors (Fig. 1a). A two-dimensional hierarchical clustering analysis of the 5214 probes revealed a clear sorting of tumors and NTLs, indicating a substantial difference in DNA methylation profiles between the tumor and non-tumor samples (Fig. 1b). With these differentially methylated probes, we investigated their regional distribution in the gene context, CpG- island neighborhood and chromosome, respectively. The gene context regions of the hyper- or hypomethylated CpG sites were distributed similarly. As indicated in Fig. 1c, most of differentially methylated probes were located in the Gene body (29% in hypermethylated and 32% in hypomethylated). However, the CpG island-based regions of the significantly hyper- or hypomethylated CpG sites are distributed differently. 60% of the hypermethylated CpG sites are in CpG islands and that fewer are in the CpG shores (24%) and CpG shelves (4%). In contrast, just 5% of the hypomethylated CpG sites were in CpG islands, CpG shores (14%) and CpG shelves (8%). In addition, chromosome location analysis showed that the majority of CpGs with differential methylation mapped to chromosome 2 and less in other chromosomes. We also added a distribution analysis of 371,000 probes in Additional file 4: Figure S4, and we compared the distribution of the differentially methylated probes and the overall probes in the genomic context. Compared to the overall distribution of all probes, the differentially methylated probes are distributed differently just in the CpG islands, 31% of the total probes located on the CpG island, 60% of the hypermethylated probes were located on the CpG island, and just 5% of the hypomethylated probes were located on the CpG island.

### Identification of potentially functionally relevant DNA methylation changes in LUSC

**Fig. 2:**
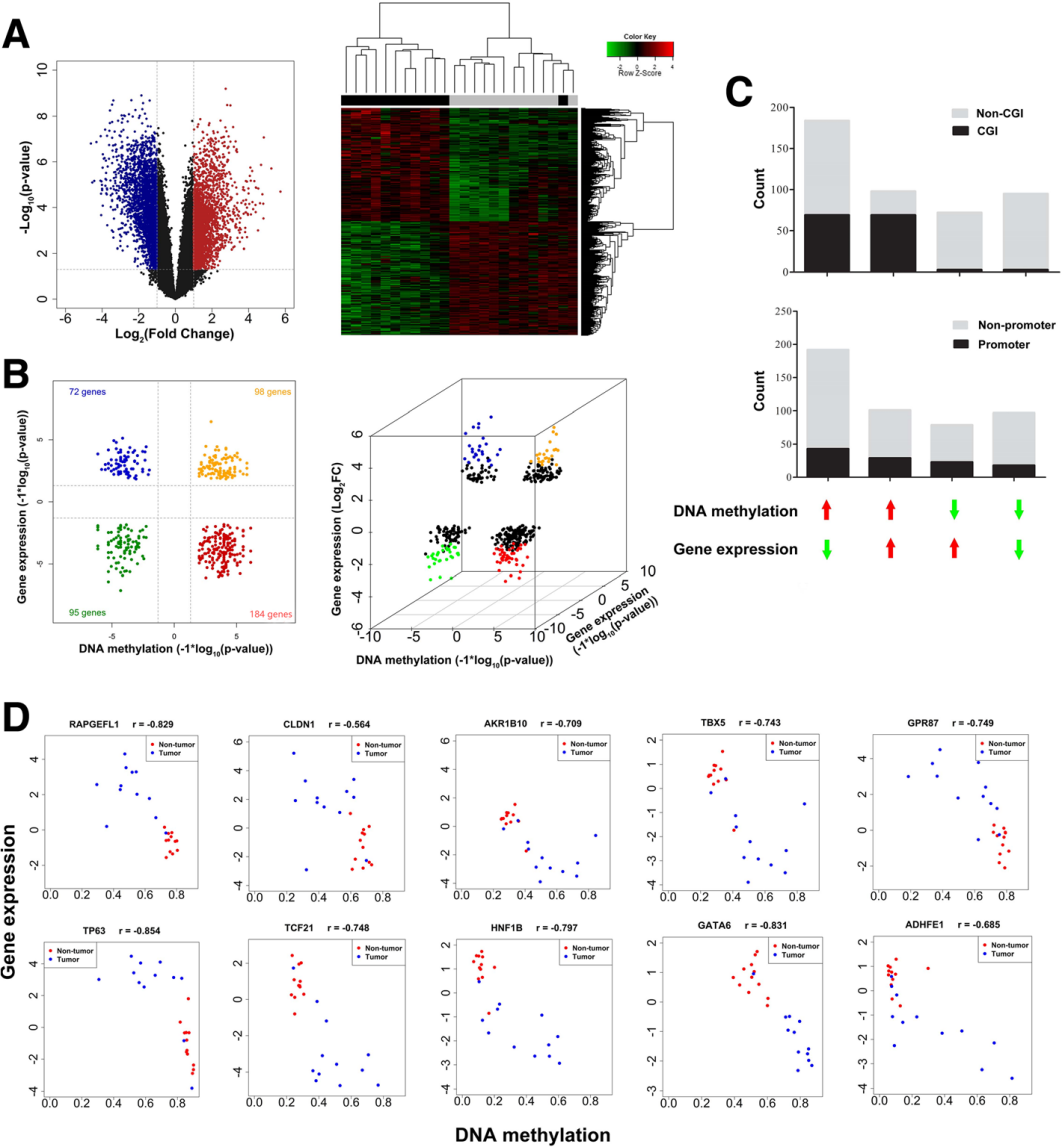
Identification of genes showing coordinately changed DNA methylation and gene expression. **a** Volcano plot and two-dimensional hierarchical clustering of the differential mRNA expression analysis. Vertical dotted lines: fold change ≥2 or ≤2; Horizontal dotted line: the significance cutoff (FDR *p*-value = 0.05). Two-dimensional hierarchical clustering was performed using 4687 probes corresponding to 3635 genes across all samples (*n* = 24). **b** Starburst plot integrating differential DNA methylation and gene expression analyses. Vertical dotted lines: the significance cutoff (FDR p-value = 0.05); Horizontal dotted line: the significance cutoff (FDR p-value = 0.05). Three-dimensional starburst plot of 123 genes, integrating significant changes in DNA methylation (x-axis) and gene expression (y-axis), with a mean twofold or greater change in gene expression (z-axis). Indicated are genes that are hypermethylated and down-regulated in tumors (red); hypomethylated and up-regulated in tumors (blue); hypermethylated and up-regulated in tumors (orange); or hypomethylated and down-regulated in tumors (green). **c** Gene distribution in CpG islands and promoter region exhibiting hyper-or hypomethylation and up- or down-regulation. **d** Correlation plots of DNA methylation versus gene expression in tumors and normal tissues for selected genes. x-axis: DNA methylation level (*β* value), y-axis: mRNA expression level, r: correlation coefficient

To identify the potential functionally relevant methylation changes, we further selected 12 paired cancer and adjacent NTL tissue to detect the genome-scale mRNA expression profiles. Corrected *P*-value <0.05 and absolute fold change >2 were used to identify differentially expressed mRNAs, 3635 genes were identified to be differentially expressed. We performed an exploratory two-dimensional hierarchical clustering of the differentially expressed probes, the mRNA expression profiles of tumors and NTL resulted in separate clusters (Fig. 2a). After integrating analysis of differentially methylated genes (DMGs) and differentially expressed genes (DEGs), we identified 449 aberrantly methylated genes accompanied with altered expression. Of these, 184 genes were statistically significantly hypermethylated and down-regulated (41%), 72 genes (16%) were significantly hypomethylated and up-regulated, while 98 genes (22%) were significantly hypermethylated and up-regulated, 95 genes (21%) were significantly hypomethylated and down-regulated. To identify genes with the greatest changes, we further set up a fourfold cutoff to the average change in gene expression (Fig. 2b), the results show that 44 genes were coordinately hypermethylated and downregulated in tumors, and 26 genes were coordinately hypomethylated and up-regulated (Additional file 2: Table S3). We next asked whether the different groups of genes were associated with CpG islands or promoter regions methylation. As indicated in Fig. 2c, we found no statistically significant difference between groups whether or not the probes were located in the promoter region (*P* > 0.05). However, there were significant differences between groups with different locations of probes in CpG island (*P* < 0.01), hypermethylated genes were gathered at CpG island. To further investigate the relationships between DNA methylation and gene expression, we selected ten genes for verification. Scatter plot demonstrated that these probes showed an inverse correlation of methylation with expression in tumor versus matched NTL, the Spearman correlation coefficient values for these ten genes were r_*RAPGEFL1*_ = −0.829, r_*CLDN1*_ = −0.564, r_*AKR1B10*_ = −0.709, r_*TP63*_ = −0.854, r_*TBX5*_ = −0.743, r_*TCF21*_ = −0.748, r_*ADHFE1*_ = −0.685, r_*GATA6*_ = −0.831, r_*GPR87*_ = −0.749, and r_*HNF1B*_ = −0.797, respectively (Fig. 2d).

**Fig. 3:**
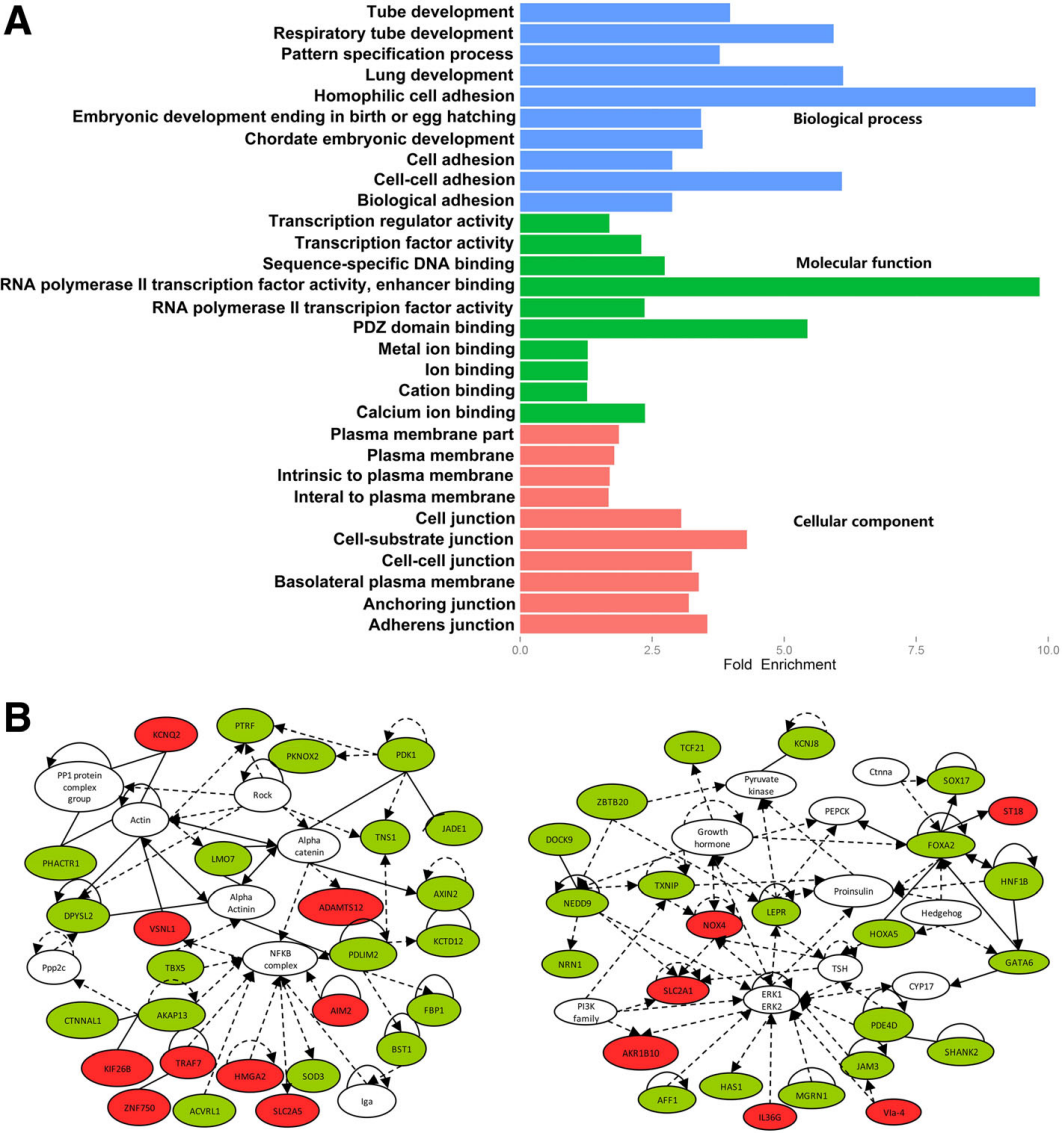
GO and pathway analysis of significant DNA methylation changes associated with significant inverse gene expression changes. **a** The top ten significantly enriched GO categories were calculated. Blue: Biological process; Green: Molecular function; Red: Cellular component. **b** Gene networks identified through integrative pathways analysis of the negatively correlated genes. Red: the hypomethylated and up-regulated genes in tumor, Green: hypermethylated and down-regulated genes in tumors, Solid lines: direct interaction, Dashed lines: indirect interaction

To study biological functions of the 70 negatively correlated genes, Gene Ontology (GO) analysis was performed. In terms of the biological processes, most of the genes were related to development and adhesion. 7 of the top 10 categories of molecular function were related to protein binding, while cellular component mostly involved the plasma membrane and cell junction (Fig. 3a). Gene network analysis was further conducted using Ingenuity Pathways Analysis (IPA), we found that top two gene networks might be affected by the aberrant DNA methylation of the 256 negative correlation genes (Fig. 3b). Prominent in the first network were PP1 protein complex members, actin gene family, and NFKB signaling pathway members. The second network was composed primarily of genes regulated by the ERK family, as well as the regulation of PIK3 complex members and Hedgehog signaling pathway members. Genes involved in the gene networks were associated with tissue morphology, organismal development, respiratory disease, cell death and survival.

### Overlap analysis

**Fig. 4:**
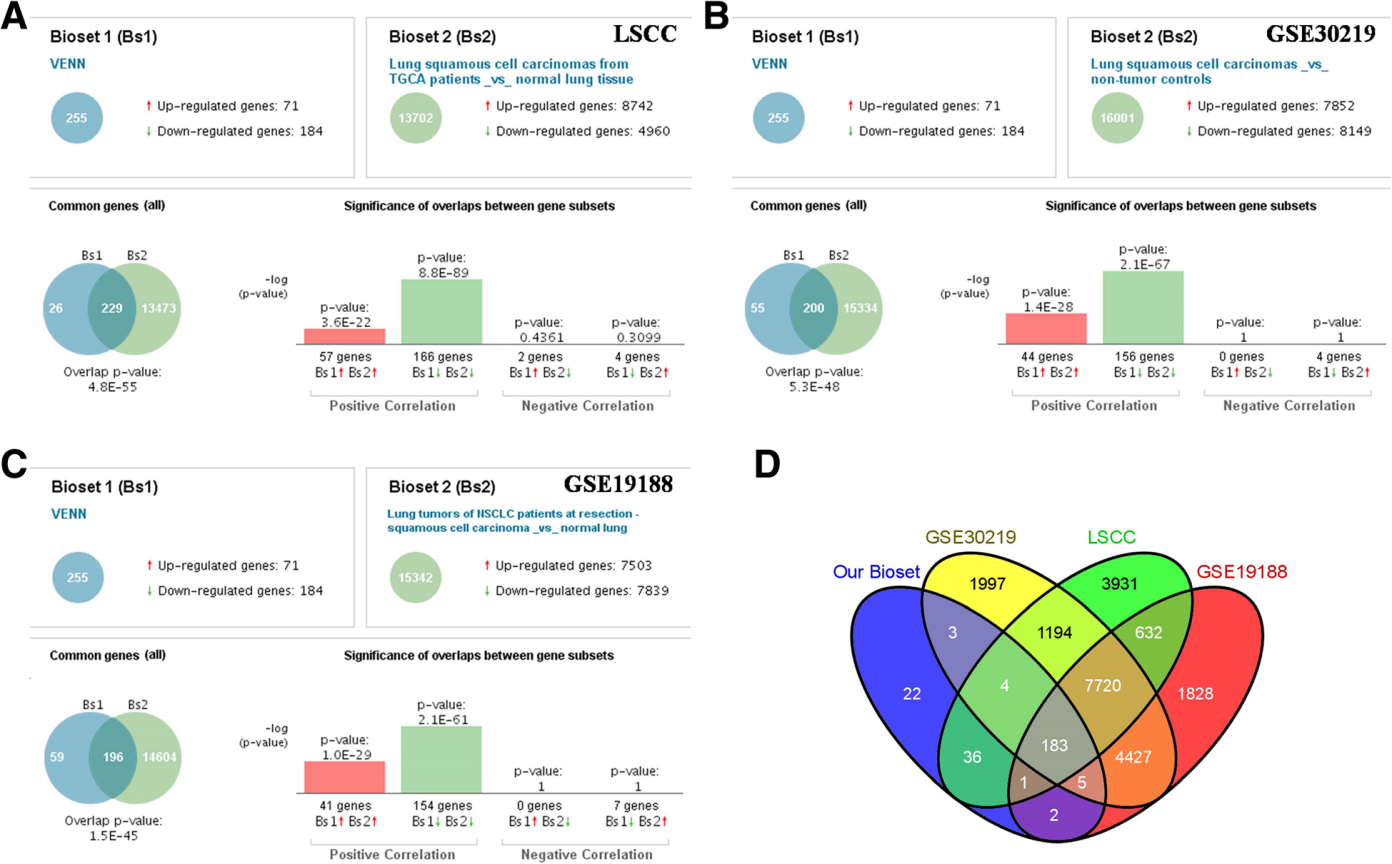
Overlap analysis between our study and other studies. Three most highly correlated NextBio biosets: LSCC(**a**), GSE30219(**b**), GSE19188(**c**). **d** Venn diagram of NextBio analysis showing the overlap of our bioset with the three most highly correlated NextBio biosets

To compare our study with other investigations, we employed the NextBio database (http://www.nextbio.com) to conduct the overlap analysis. The three most highly correlated NextBio biosets (LSCC, GSE30219, GSE19188) were selected. As indicated in Fig. 4a, of the 256 genes with inverse correlations, a total of 229 genes were significantly differentially expressed in our study and TCGA dataset (LSCC), most of the genes were expressed at the same direction, while 2 up regulated genes were down regulated in the LSCC dataset, and 4 down regulated genes were up regulated in LSCC dataset. To learn more about the overlap between our microarray and GEO database, we compared current study’s differentially expressed genes with their results. As showed in Fig. 4b the two studies owned an overlap of 200 genes, just few genes were expressed at the different direction. Similar to this result, Fig. 4c showed that 196 genes were overlapped between our study and GSE19188 dataset. Taken together, there were 183 overlapping genes, highly consistent with the three previous studies on tumor and matched NTL (Fig. 4d).

### Validation of the methylation biomarkers for LUSC diagnosis

**Fig. 5:**
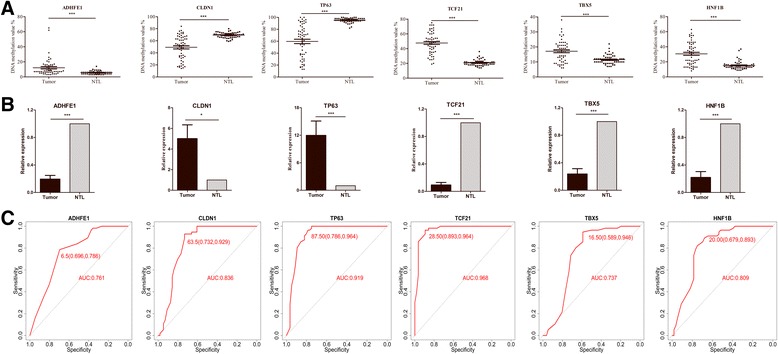
Validation of selected methylation biomarkers. **a** Clinical validation of DNA methylation levels of selected genes in paired LUSC and adjacent NTL tissue by using pyrosequencing. **b** Clinical validation of mRNA expression of selected genes in paired LUSC and adjacent NTL tissue by using qRT-PCR. **c** ROC curves and area under the curve (AUC) for the candidate genes. Sensitivity, Specificity and the optimal cut-off values were marked in the figures. *** corresponds to *p* < 0.001; ** *p* < 0.01 and * *p* < 0.05

To confirm our previous results, we selected six genes (CLDN1, TP63, TBX5, TCF21, ADHFE1 and HNF1B) for further validation in another independent 56 paired LUSC and adjacent NTL tissues. The clinical characteristics of this cohort were summarized in Table 1 **.** DNA methylation was detected by using pyrosequencing, mRNA expression was identified by using realtime PCR. As indicated in Fig. 5a and b, the results were consistent with our high-throughput analysis, and we found two hypomethylation and up-regulated expression genes, four hypermethylation and down-regulated expression genes. Next, receiver operating characteristics (ROC) analysis was performed to assess the diagnostic value of each individual biomarker to detect LUSC. Areas under the ROC curve (AUC) of tumor and NTL group were significantly different (*P* < 0.01) for all six genes with the following values AUC_*CLDN1*_ = 0.836, AUC_*TP63*_ = 0.919, AUC_*TBX5*_ = 0.737, AUC_*TCF21*_ = 0.968, AUC_*ADHFE1*_ = 0.761 and AUC_*HNF1B*_ = 0.809 (Fig. 5c). Considering that our study was limited by the number of patients, we expanded the sample size to further validation by using the Cancer Genome Atlas (TCGA) database. A total of 343 LUSC patients and 39 NTL tissue samples were selected (Additional file 2: Table S4). The methylation levels of the six selected genes were similar to those of our clinical validation cohort with significant differences between tumor and NTL (Additional file 5: Figure S3A), suggesting that the methylation statuses of the six selected biomarkers are a common feature for LUSC. Then, we performed ROC analysis to assess the performance of each individual biomarker to detect LUSC. Importantly, all the genes showed significant difference (*P* < 0.01) in AUC (AUC_*CLDN1*_ = 0.919, AUC_*TP63*_ = 0.958, AUC_*TBX5*_ = 0.984, AUC_*TCF21*_ = 0.985, AUC_*ADHFE1*_ = 0.852 and AUC_*HNF1B*_ = 0.908), suggesting that they could be suitable as potential predictive biomarkers for LUSC diagnosis (Additional file 5: Figure S3B). Details of the CGs dinucleotides for these six genes are listed in Additional file 2: Table S5.

### Subclassification of LUSC by methylation patterns

**Fig. 6:**
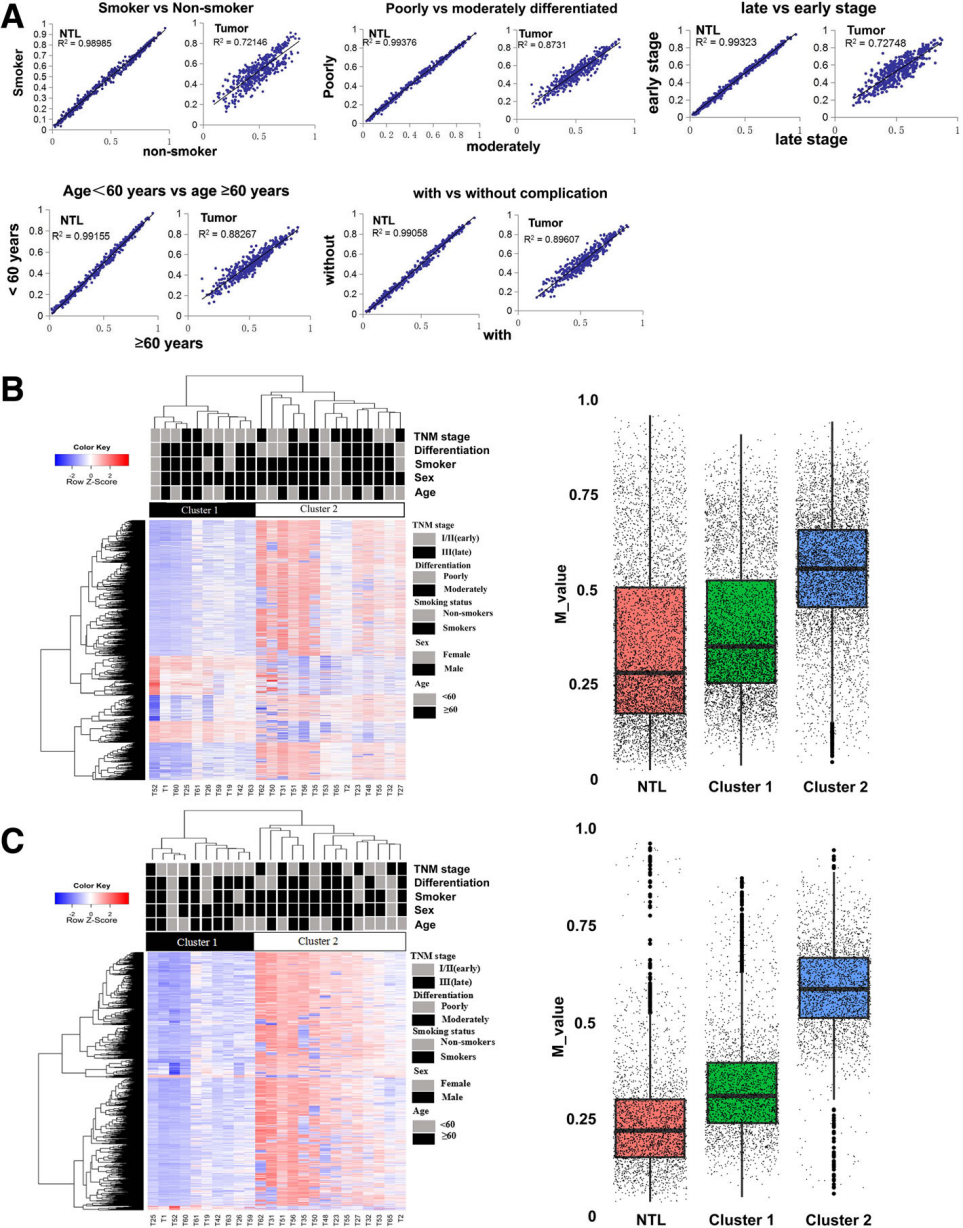
Subgroup analysis of DNA methylation between LUSC and NTL. **a** Subgroup analysis of DNA methylation between LUSC and NTL according to smoking status, differentiation, TNM stage, age and complications. The correlation coefficient was given in the left corner. **b** Two-dimensional hierarchical clustering of all the significantly differently methylated probes in tumors was performed (n = 24). DNA methylation levels of Cluster 1, Custer 2 and NTL are shown using M-values. Note that methylation levels were significantly higher in Cluster 2 than in Cluster 1, although those of Cluster 1 were higher than those of NTL. **c** Two-dimensional hierarchical clustering of the 2470 differentially methylated probes (on the CpG island) was performed, two distinct clusters were identified. DNA methylation levels of Cluster 1, Custer 2 and NTL are shown using M-values. Note that methylation levels were significantly higher in Cluster 2 than in Cluster 1

Finally, to explore the effect of clinical pathological features on DNA methylation, we performed correlation studies based on the stratification of clinical characteristics. Patients were divided into two groups according to each of the following five factors: age (<60 or ≥60 years old), smoking status (smokers or non-smokers), differentiation (poorly or moderately), complication (with or without) and TNM stages (I/II or III). The results showed that all factors mentioned above were highly associated with DNA methylation,respectively; and associations of each factor with DNA methlylation were more evident in NTL groups relative to tumor group (Fig. 6a). Then, to identify squamous cell lung cancer DNA methylation-based subclasses, we used the 5214 differentially methylated probes to perform an unsupervised hierarchical clustering. 24 tumors were clearly divided into two independent categories: Cluster1 (*n* = 10) and Cluster 2 (*n* = 14), Cluster 1 tumors were significantly hypomethylated as compared with Cluster 2 tumors (Fig. 6b). We also used the 2470 differentially methylated probes on the CpG island to perform an unsupervised hierarchical clustering, which suggested more significant differences between the two tumor subclusters (Fig. 6c).

## Discussion

In the present study, we investigated the genome-wide DNA methylation patterns in 24 paired LUSC and adjacent NTL tissues by microarray, and identified 5214 probes showing significantly differential DNA methylation in cancer tissues. By integrating DNA methylation and mRNA expression data, 449 aberrantly methylated genes accompanied with altered expression were identified. GO analysis of these genes indicated that the most significantly related terms were development and binding. Pathway analysis highlighted many pathways which were closely related to the carcinogenesis of LUSC, such as ERK family, NFKB signaling pathway, Hedgehog signaling pathway, providing new clues for understanding the molecular mechanisms of LUSC pathogenesis. To verify the results of high-throughput screening, we used 56 paired independent tissues for clinical validation by pyrosequencing. Subsequently, another 343 tumor tissues from the TCGA were utilized for further validation. Then, we identified a panel of DNA methylation biomarkers in LUSC. Furthermore, we performed ROC analysis to assess the performance of each biomarker, results suggested that they could serve as potential predictive biomarkers for LUSC diagnosis. Finally, hierarchical clustering analysis of the DNA methylation data identified two tumor subgroups, one of which showed increased DNA methylation.

The pattern of DNA methylation in some certain types of cancers has been investigated including NSCLC [12]. DNA methylation analysis of cell-free blood samples has a substantial potential to serve as a minimally invasive tool for early diagnosis and clinical monitoring of cancer. Wielscher et al. found a model of four genes (HOXD10, PAX9, PTPRN2 and STAG3) that were able to differentiate lung cancer from controls, fibrotic ILD, and COPD [19]. NSCLC comprise multiple distinct biologic groups (such as epithelial-like NSCLCs and mesenchymal-like NSCLCs) with different prognoses. Walter et al. showed that patterns of DNA methylation can divide NSCLCs into these two phenotypically distinct subtypes of tumors and provide proof of principle that differences in DNA methylation can be used as a platform for predictive biomarker discovery and development [20]. Another research identified differentially methylated genes by comparing the global DNA methylation patterns between lung adenocarcinoma samples from smokers and nonsmokers. Their study provides an insightful perspective on smoking-associated DNA methylation and its role in tumorigenesis of the lung [21]. We investigated the genome-scale DNA methylation profile in LUSC and identified 5214 differentially methylated probes. Certain aberrantly methylated genes that were found in our study were also reported in previous other studies, which supported the result for each other. One of the previously reported methylated genes was *SOX17*, a canonical WNT antagonist previously shown functionally hypermethylated in breast, colorectal and lung cancers [22–24]. In our study, *SOX17* was also a hypermethylated and down-regulated expression gene. Another example was *WIF1*, an extracellular antagonist that acts by binding to Wnt ligands. According to current findings, WIF1 promoter methylation was a frequent event as an epigenetic field manner and could be considered as a useful prognostic marker for adenocarcinoma patients [25]. In agreement with this report, our results also showed that WIF1 expression was down-regulated by promoter hypermethylation in LUSC. However, most of the differentially methylated genes identified in this study were novel. The top hypomethylated and up-regulated gene, aldo-keto reductase family 1 member B10 (AKR1B10), has been demonstrated previously to be specifically up-regulated in smoking-associated cancers such as squamous cell carcinoma and adenocarcinoma [26]. B-arrestin-1 (ARRB1), a scaffolding protein involved in the desensitization of signals arising from activated G-protein-coupled receptors, has been shown to play a role in invasion and proliferation of cancer cells, including nicotine-induced proliferation of NSCLC [27].

We selected several genes for clinical validation, and six genes (CLDN1, TP63, TBX5, TCF21, ADHFE1 and HNF1B) were identified. CLDN1 serves as an oncogene or a tumor suppressor in a tissue-specific manner. There is a significant correlation between down regulation of CLDN1 expression and methylation of its promoter CpG-island in estrogen receptor positive breast cancer [28, 29]. While our results suggested that hypomethylation might contribute to the upregulation of CLDN1 in LUSC and CLDN1 overexpression may play a role in the pathogenesis of LUSC. For the other genes, p63 was reported to be overexpressed in many tumors especially in LUSC [30–32], whereas adenocarcinoma and small cell carcinomas were almost all p63 low expression. TBX5 is a member of a phylogenetically conserved family of genes involved in the regulation of development,it is a novel functional tumor suppressor gene inactivated by promoter methylation in colon cancer [33]. TCF21 hypermethylation and reduced protein expression are ubiquitous in NSCLC [34]. TCF21 is expressed in normal lung airway epithelial cells, however, it is aberrantly methylated and silenced in the majority of head and neck squamous cell carcinomas and in NSCLC [35]. ADHFE1, a member of the group III metal dependent alcohol dehydrogenase family. The hypermethylation of ADHFE1 has recently been reported to be associated with colorectal cancer differentiation [36]. We performed receiver operating characteristics (ROC) analysis to assess the performance of each individual biomarker to detect LUSC. Our results suggested a strong diagnostic potential for these markers, and we hoped that they are potentially applicable in improving early LUSC diagnosis.

To further validate our findings, we asked if the findings of the three most highly correlated investigations (LSCC, GSE30219, GSE19188) are consistent with our results. For the overlapped genes, our study and the previous investigations had high consistency with each other. The high reliability and reproducibility of the microarray technology in identifying the six genes are essential for its application in discovering the clinical biomarkers.

This study lays a foundation for the diagnosis, treatment and functional research of LUSC. However, there are some limitations in this study. We just examined the methylation of the target genes in tissue samples. In the future, we will also detect the methylation of these biomarkers in minimally/non-invasive samples.

## Conclusions

In summary, we have identified and independently validated a powerful epigenetic signature of LUSC in tissue samples. We also described the clinicopathological characteristics of distinct molecular LUSC subgroups. The current study demonstrated that differences in genome-wide DNA methylation and gene expression patterns exist between LUSC and NTL. Our results suggested that DNA methylation plays critical roles in lung tumorigenesis and may potentially be proposed as a diagnostic biomarker.

## Additional files

Additional file 1: Figure S1. Sketch and pipeline of the study design. (TIFF 2547 kb)

Additional file 2: Supplementary Materials. This docx file contains all supplementary tables. (DOCX 36 kb)

Additional file 3: Figure S2. Genomic context of CpG methylation. (**A**) The overall distribution of methylation sites in tumor versus NTL (**B**) A schematic diagram of CpGs depicts their genomic context relative to the nearest CpG island (top) or gene (bottom). (**C, D**) Density distribution of methylation probes in the CpG island-based regions and the gene-based regions. The x-axis is the meanβvalue in different regions. The y-axis is the signal density. The blue line is tumor, the red line is non-tumor. Transcription start site (TSS) 1500, TSS200, 5’untranslated region (UTR), and 3′ UTR. (TIFF 2060 kb)

Additional file 4: Figure S4. The distribution of 371,000 probes in gene context, CpG-site neighborhood and chromosome, respectively. TSS: transcription start site, UTR: untranslated region, Chr: chromosome. (TIFF 9799 kb)

Additional file 5: Figure S3. Validation of the methylation biomarkers using 343 LUSC and 39 NTL tissue from TCGA database. (**A**) Validation of selected methylation biomarkers. ***corresponds to *P* < 0.01. (**B**) ROC curves and area under the curve (AUC) with 95% confidence intervals for the candidate genes. (TIFF 5194 kb)

## Abbreviations

AUC: Area under the curve
CGI: CpG island
CI: Confidence intervals
DEGs: Differentially expressed genes
DMGs: Differentially methylated genes
GO: Gene Ontology
IPA: Ingenuity Pathways Analysis
LUSC: Squamous cell lung cancer
NSCLC: Non-small cell lung cancer
NTL: Non-tumor lung
ROC: Receiver operating characteristics
TCGA: The Cancer Genome Atlas
TSS: Transcription start site
UTR: Untranslated region

## References

[1] Siegel RL, Miller KD, Jemal A (2015). Cancer statistics, 2015. CA Cancer J Clin.

[2] Liloglou T, Bediaga NG, Brown BR, Field JK, Davies MP (2014). Epigenetic biomarkers in lung cancer. Cancer Lett.

[3] Barros SP, Offenbacher S (2009). Epigenetics: connecting environment and genotype to phenotype and disease. J Dent Res.

[4] Heyn H, Esteller M (2012). DNA methylation profiling in the clinic: applications and challenges. Nat Rev Genet.

[5] Balgkouranidou I, Liloglou T, Lianidou ES (2013). Lung cancer epigenetics: emerging biomarkers. Biomark Med.

[6] Dai Z, Lakshmanan RR, Zhu WG, Smiraglia DJ, Rush LJ, Fruhwald MC, Brena RM, Li B, Wright FA, Ross P (2001). Global methylation profiling of lung cancer identifies novel methylated genes. Neoplasia.

[7] Diaz-Lagares A, Mendez-Gonzalez J, Hervas D, Saigi M, Pajares MJ, Garcia D, Crujeiras AB, Pio R, Montuenga LM, Zulueta J (2016). A novel epigenetic signature for early diagnosis in lung cancer. Clin Cancer Res.

[8] Konecny M, Markus J, Waczulikova I, Dolesova L, Kozlova R, Repiska V, Novosadova H, Majer I (2016). The value of SHOX2 methylation test in peripheral blood samples used for the differential diagnosis of lung cancer and other lung disorders. Neoplasma.

[9] Xiao P, Chen JR, Zhou F, Lu CX, Yang Q, Tao GH, Tao YJ, Chen JL (2014). Methylation of P16 in exhaled breath condensate for diagnosis of non-small cell lung cancer. Lung Cancer.

[10] Hwang JA1, Lee BB, Kim Y, Park SE, Heo K, Hong SH, Kim YH, Han J, Shim YM, Lee YS (2013). HOXA11 hypermethylation is associated with progression of non-small cell lung cancer. Oncotarget.

[11] Balgkouranidou I, Chimonidou M, Milaki G, Tsaroucha E, Kakolyris S, Georgoulias V, Lianidou E (2016). SOX17 promoter methylation in plasma circulating tumor DNA of patients with non-small cell lung cancer. Clin Chem Lab Med.

[12] Mullapudi N, Ye B, Suzuki M, Fazzari M, Han W, Shi MK, Marquardt G, Lin J, Wang T, Keller S (2015). Genome wide Methylome alterations in lung cancer. PLoS One.

[13] Gandara DR, Hammerman PS, Sos ML, Lara PN, Hirsch FR (2015). Squamous cell lung cancer: from tumor genomics to cancer therapeutics. Clin Cancer Res.

[14] Wang T, Zhang L, Tian P, Tian S (2017). Identification of differentially-expressed genes between early-stage adenocarcinoma and squamous cell carcinoma lung cancer using meta-analysis methods. Oncol Lett.

[15] Zhang W, Spector TD, Deloukas P, Bell JT, Engelhardt BE (2015). Predicting genome-wide DNA methylation using methylation marks, genomic position, and DNA regulatory elements. Genome Biol.

[16] Wang Y, Qian C-Y, Li X-P, Zhang Y, He H, Wang J, Chen J, Cui J-J, Liu R, Zhou H (2015). Genome-scale long noncoding RNA expression pattern in squamous cell lung cancer. Sci Rep.

[17] Shi Y-X, Yin J-Y, Shen Y, Zhang W, Zhou H-H, Liu Z-Q (2017). Genome-scale analysis identifies NEK2, DLGAP5 and ECT2 as promising diagnostic and prognostic biomarkers in human lung cancer. Sci Rep.

[18] Liu R, Guo CX, Zhou HH (2015). Network-based approach to identify prognostic biomarkers for estrogen receptor-positive breast cancer treatment with tamoxifen. Cancer Biol Ther.

[19] Wielscher M, Vierlinger K, Kegler U, Ziesche R, Gsur A, Weinhäusel A (2015). Diagnostic performance of plasma DNA Methylation profiles in lung cancer, pulmonary fibrosis and COPD. EBioMedicine.

[20] Walter K, Holcomb T, Januario T, Du P, Evangelista M, Kartha N, Iniguez L, Soriano R, Huw L, Stern H (2012). DNA Methylation profiling defines clinically relevant biological subsets of non-small cell lung cancer. Clin Cancer Res.

[21] Lu S, Tan Q, Wang G, Huang J, Ding Z, Luo Q, Mok T, Tao Q. Epigenomic analysis of lung adenocarcinoma reveals novel DNA methylation patterns associated with smoking. OncoTargets Therapy. 2013;1471 10.2147/OTT.S51041 PMC3818101 24204162

[22] Zhang W, Glockner SC, Guo M, Machida EO, Wang DH, Easwaran H, Van Neste L, Herman JG, Schuebel KE, Watkins DN et al: Epigenetic inactivation of the canonical Wnt antagonist SRY-box containing gene 17 in colorectal cancer. Cancer Res 2008, 68(8):2764-2772. 10.1158/0008-5472.CAN-07-6349 PMC2823123 18413743

[23] Fu DY, Wang ZM, Li C, Wang BL, Shen ZZ, Huang W, Shao ZM (2010). Sox17, the canonical Wnt antagonist, is epigenetically inactivated by promoter methylation in human breast cancer. Breast Cancer Res Treat.

[24] Yin D, Jia Y, Yu Y, Brock MV, Herman JG, Guo M (2012). SOX17 Methylation Inhibits Its Antagonism of Wnt Signaling Pathway in Lung Cancer. Discov Med.

[25] Lee SM, Park JY, Kim DS (2013). Wif1 hypermethylation as unfavorable prognosis of non-small cell lung cancers with EGFR mutation. Mol Cells.

[26] Penning TM. AKR1B10: a new diagnostic marker of non–small cell lung carcinoma in smokers. Clin Cancer Res. 2005; 10.1158/1078-0432.CCR-05-0071 15755988

[27] Perumal D, Pillai S, Nguyen J, Schaal C, Coppola D, Srikumar P (2014). Chellappan: Nicotinic acetylcholine receptors induce c-Kit ligand/Stem Cell Factor and promote stemness in an ARRB1/β-arrestin-1 dependent manner in NSCLC. Oncotarget.

[28] Di Cello F, Cope L, Li H, Jeschke J, Wang W, Baylin SB, Zahnow CA: Methylation of the claudin 1 promoter is associated with loss of expression in estrogen receptor positive breast cancer. PLoS One 2013, 8(7):e68630. 10.1371/journal.pone.0068630 PMC3701071 23844228

[29] Ogoshi K, Hashimoto S-i, Nakatani Y, Qu W, Oshima K, Tokunaga K, Sugano S, Hattori M, Morishita S, Matsushima K: Genome-wide profiling of DNA methylation in human cancer cells. Genomics 2011, 98(4):280-287. 10.1016/j.ygeno.2011.07.003 21821115

[30] Nobre AR, Albergaria A, Schmitt F (2013). p40: a p63 isoform useful for lung cancer diagnosis - a review of the physiological and pathological role of p63. Acta Cytol.

[31] Wang BY, Gil J, Kaufman D, Gan L, Kohtz DS, Burstein DE (2002). p63 in pulmonary epithelium, pulmonary squamous neoplasms, and other pulmonary tumors. Hum Pathol.

[32] Cancer Genome Atlas Research N (2012). Comprehensive genomic characterization of squamous cell lung cancers. Nature.

[33] Yu J, Ma X, Cheung KF, Li X, Tian L, Wang S, Wu CW, Wu WK, He M, Wang M (2010). Epigenetic inactivation of T-box transcription factor 5, a novel tumor suppressor gene, is associated with colon cancer. Oncogene.

[34] Richards KL, Zhang B, Sun M, Dong W, Churchill J, Bachinski LL, Wilson CD, Baggerly KA, Yin G, Hayes DN (2011). Methylation of the candidate biomarker TCF21 is very frequent across a spectrum of early-stage nonsmall cell lung cancers. Cancer.

[35] Smith LT, Lin M, Brena RM, Lang JC, Schuller DE, Otterson GA, Morrison CD, Smiraglia DJ, Plass C (2006). Epigenetic regulation of the tumor suppressor gene TCF21 on 6q23-q24 in lung and head and neck cancer. Proc Natl Acad Sci.

[36] Moon JW, Lee SK, Lee YW, Lee JO, Kim N, Lee HJ, Seo JS, Kim J, Kim HS, Park SH (2014). Alcohol induces cell proliferation via hypermethylation of ADHFE1 in colorectal cancer cells. BMC Cancer.

