# Supplementary figures and images for: Genome-wide DNA methylation profiling reveals novel epigenetic signatures in squamous cell lung cancer
Source: BMC Genomics. 2017 Nov 23;18:901. doi: 10.1186/s12864-017-4223-3 (PMC5701423; doi:10.1186/s12864-017-4223-3)

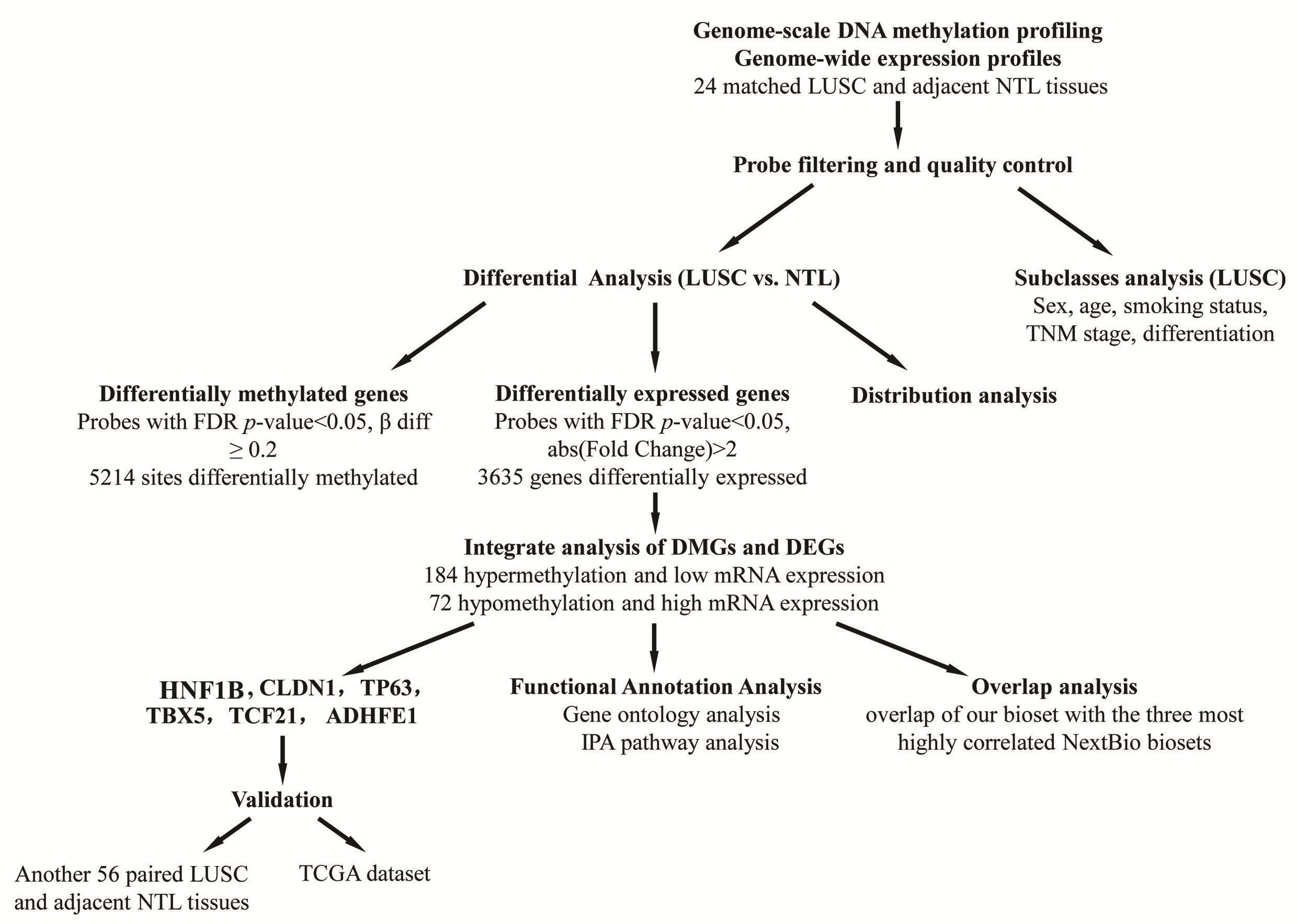

Supplement: Supplementary file 1 — Sketch and pipeline of the study design. (TIFF 2547 kb) [file 12864_2017_4223_MOESM1_ESM.tif]

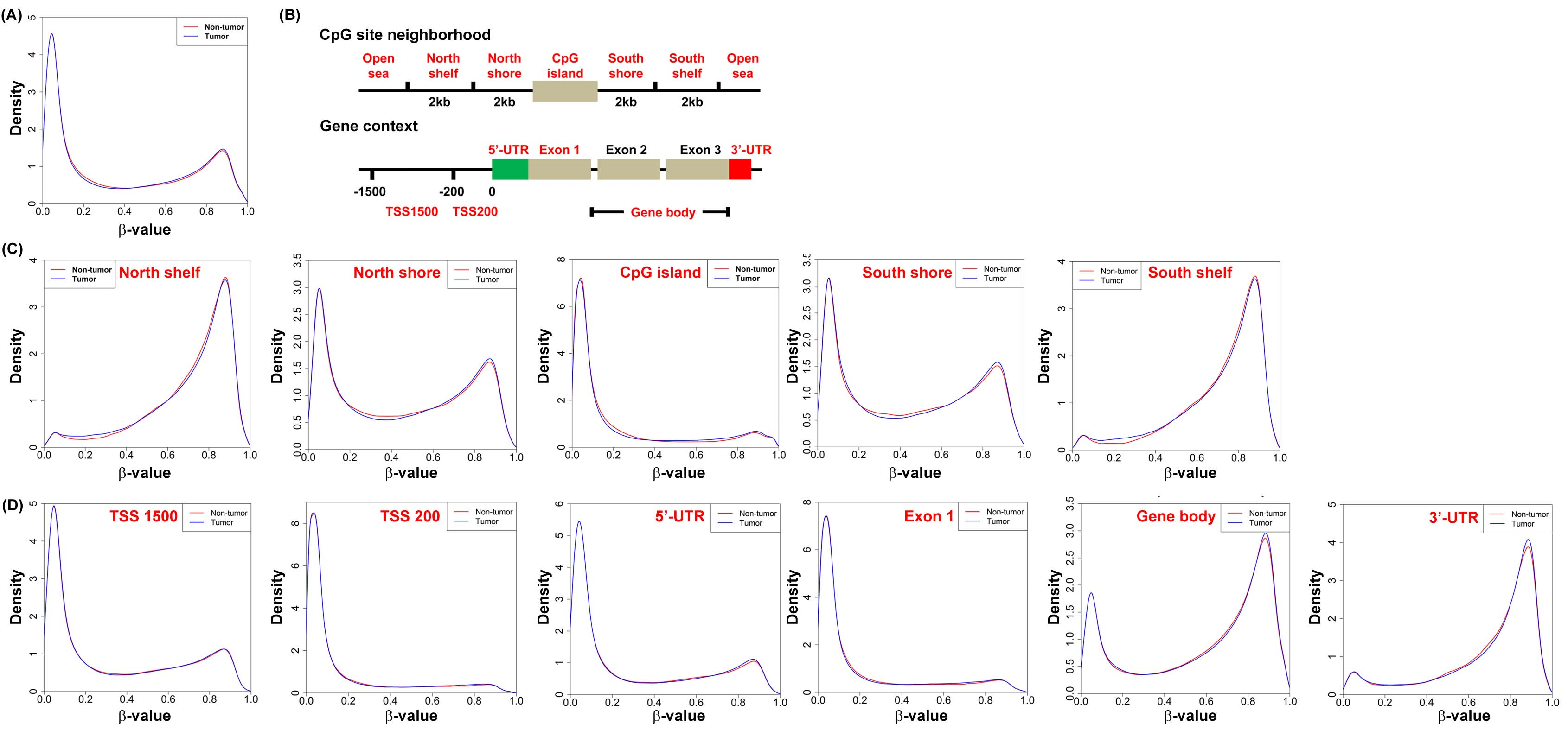

Supplement: Supplementary file 3 — Genomic context of CpG methylation. (A) The overall distribution of methylation sites in tumor versus NTL (B) A schematic diagram of CpGs depicts their genomic context relative to the nearest CpG island (top) or gene (bottom). (C, D) Density distribution of methylation probes in the CpG island-based regions and the gene-based regions. The x-axis is the meanβvalue in different regions. The y-axis is the signal density. The blue line is tumor, the red line is non-tumor. Transcription start site (TSS) 1500, TSS200, 5’untranslated region (UTR), and 3′ UTR. (TIFF 2060 kb) [file 12864_2017_4223_MOESM3_ESM.tif]

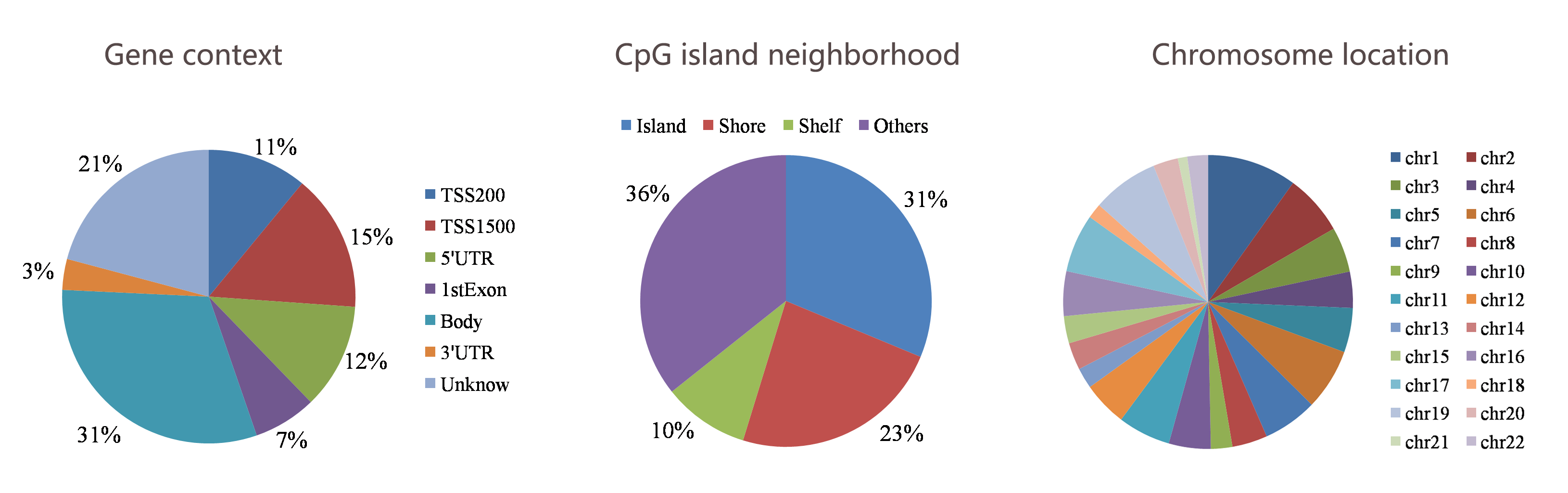

Supplement: Supplementary file 4 — The distribution of 371,000 probes in gene context, CpG-site neighborhood and chromosome, respectively. TSS: transcription start site, UTR: untranslated region, Chr: chromosome. (TIFF 9799 kb) [file 12864_2017_4223_MOESM4_ESM.tif]

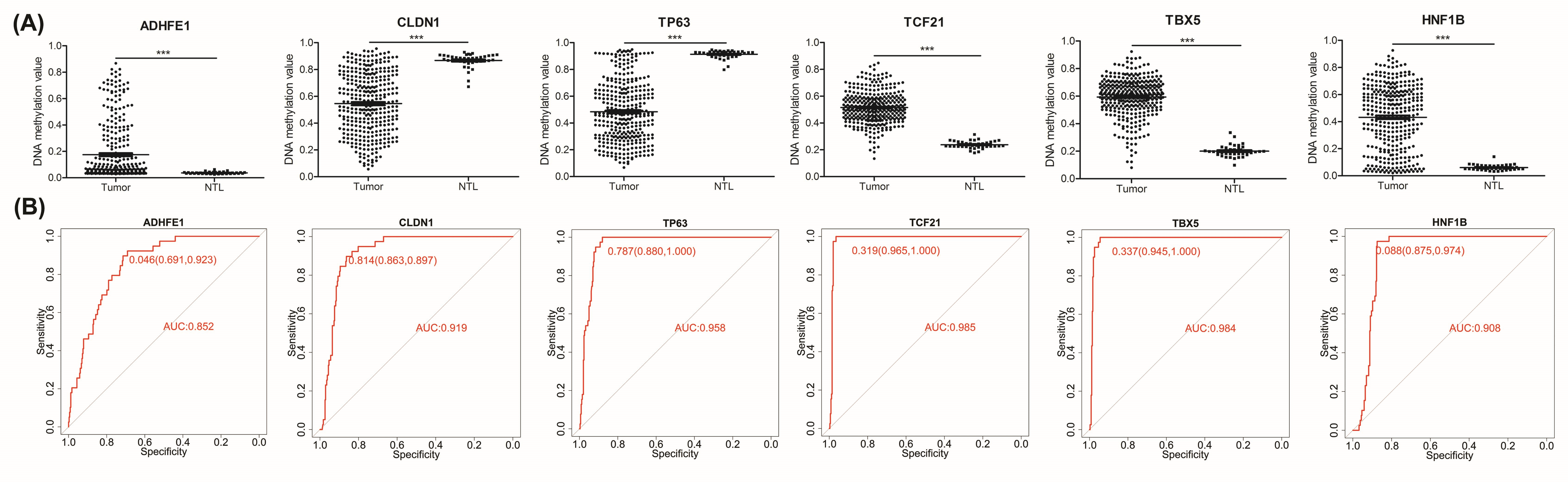

Supplement: Supplementary file 5 — Validation of the methylation biomarkers using 343 LUSC and 39 NTL tissue from TCGA database. (A) Validation of selected methylation biomarkers. ***corresponds to P < 0.01. (B) ROC curves and area under the curve (AUC) with 95% confidence intervals for the candidate genes. (TIFF 5194 kb) [file 12864_2017_4223_MOESM5_ESM.tif]
