# Supplementary material for: Genome-wide DNA methylation profiling reveals novel epigenetic signatures in squamous cell lung cancer
Source: BMC Genomics. 2017 Nov 23;18:901. doi: 10.1186/s12864-017-4223-3 (PMC5701423; doi:10.1186/s12864-017-4223-3)
Supplement: Supplementary file 2 — This docx file contains all supplementary tables. (DOCX 36 kb) [file 12864_2017_4223_MOESM2_ESM.docx]

Supplementary Materials

Supplementary Table 1 Primers for pyrosequencing and qRT-PCR

| Gene | Probe ID | Primer |
| --- | --- | --- |
| Pyrosequencing | | |
| CLDN1 | cg03601836 | F: TTGGTGGGAGTAAATATTATTGTTTAGGT |
|  |  | R: [Biot]AAAAACCCTTAACTCACAAATATTCT |
|  |  | S: TTTTTGGGTTTTATTTAAAAGTATT |
| TP63 | cg06520450 | F: TGGATTGGATAGGTAAAGAGAAGAGT |
|  |  | R: [Biot] AATTTCCTAATATATACCCAACTATAAACA |
|  |  | S: ATAGGTAAAGAGAAGAGTT |
| TBX5 | cg26196480 | F: GGAGGAGTTTTGGGTAAATGAAT |
|  |  | R: [Biot] TCCCCTCCACCACAACCCAATA |
|  |  | S: TGGTTAGGTTTGGGA |
| TCF21 | cg04692403 | F: [Biot] TAGTTAGGAGGGGAAGTAGGTT |
|  |  | R: ACACCCAAAACAAAATAATCTTAAATC |
|  |  | S: ATCTTAAATCTAAAAAAAACCTTAC |
| ADHFE1 | cg09383816 | F: TGATAAGTAAGGAGATTTAAGGTAGAAT |
|  |  | R: [Biot] CAAACCAATCCAACTCAAAACCATTTTC |
|  |  | S: TTTAAGGGTGGATGG |
| HNF1B | cg19378036 | F: [Biot] TGGATTTGTTAAGTTAGTGTTTTGTAGT |
|  |  | R: ACACCCCAACTTCCTCTA |
|  |  | S: ACCCCCAAACCTAATATCTTCTCTC |
| qRT-PCR | | |
| GAPDH |  | F: CCCATCACCATCTTCCAGGAG |
|  |  | R: GTTGTCATGGATGACCTTGGC |
| TBX5 |  | F: CTGTGGCTAAAATTCCACGAAGT |
|  |  | R: GTGATCGTCGGCAGGTACAAT |
| CLDN1 |  | F: TCTGGCTATTTTAGTTGCCACAG |
|  |  | R: AGAGAGCCTGACCAAATTCGT |
| TCF21 |  | F: TCCTGGCTAACGACAAATACGA |
|  |  | R: TTTCCCGGCCACCATAAAGG |
| TP63 |  | F: GGACCAGCAGATTCAGAACGG |
|  |  | R: AGGACACGTCGAAACTGTGC |
| ADHFE1 |  | F: TGGACTTTCACCTTCTGGGAA |
|  |  | R: GGAGAGGTTCTTGTCTGTCATCA |
| HNF1B |  | F: GAGGAGGCATTCCGGCAAAA |
|  |  | R: TGTAAAACCGACTGGCTGGTC |

F: Forward primer, R: Reverse primer, S: Sequencing primer

Supplementary Table 2 Comparison of methylation level distribution between LUSC and NTL

| Regions | Probes  (N) | mean value | | *P* value |
| --- | --- | --- | --- | --- |
|  |  | LCSC | NTL |  |
| Whole genome | 238,616 | 0.378 | 0.386 | 2.20E-16 |
|  |  |  |  |  |
| TSS1500 | 65,500 | 0.344 | 0.343 | 2.96E-08 |
|  |  |  |  |  |
| TSS200 | 47,544 | 0.184 | 0.189 | 2.20E-16 |
|  |  |  |  |  |
| 3'UTR | 14,715 | 0.7 | 0.701 | 2.20E-16 |
|  |  |  |  |  |
| 5'UTR | 50,408 | 0.314 | 0.318 | 2.20E-16 |
|  |  |  |  |  |
| body | 134,357 | 0.596 | 0.597 | 2.20E-16 |
|  |  |  |  |  |
| 1st Exon | 30,086 | 0.212 | 0.219 | 2.20E-16 |
|  |  |  |  |  |
| Island | 115,964 | 0.221 | 0.239 | 2.20E-16 |
|  |  |  |  |  |
| North shelf | 18,700 | 0.711 | 0.702 | 2.20E-16 |
|  |  |  |  |  |
| South shelf | 16,754 | 0.718 | 0.706 | 2.20E-16 |
|  |  |  |  |  |
| South shore | 38,239 | 0.443 | 0.446 | 2.20E-16 |
|  |  |  |  |  |
| North shore | 48,963 | 0.453 | 0.458 | 2.20E-16 |
|  |  |  |  |  |

Supplementary Table 3 Top hyper-or hypomethylated and up- or down-regulated genes in LUSC

| Gene symbol | Gene name | Annotation |
| --- | --- | --- |
| Top hypermethylated and down-regulated genes in LUSC | | |
| SFTA3 | Surfactant associated 3 | A protein-coding gene |
| WIF1 | WNT inhibitory factor 1 | Inhibit WNT proteins |
| TCF21 | Transcription factor 21 | Epithelial-mesenchymal interactions |
| NKX2-1 | NK2 homeobox 1 | Thyroid-specific transcription factor |
| C2orf40 | Chromosome 2 open reading frame 40 | G1 arrest, RB1 dephosphorylation and accelerated CCND1 and CCND3 proteasomal degradation |
| HSPB6 | Heat shock protein, alpha-crystallin-related, B6 | Encodes a heat shock protein |
| TNS1 | Tensin 1 | Adhesion formation， cell migration |
| ADRA1A | A1A-adrenergic receptor | Activate mitogenic responses and regulate growth and proliferation |
| HPSE2 | Heparanase 2 | Encodes a heparanase enzyme |
| C4orf31 | Chromosome 4 open reading frame 31 | Neuron-derived neurotrophic factor |
| UPK3B | Uroplakin 3B | Component of the asymmetric unit membrane |
| PCDHA12 | Protocadherin alpha 12 | Calcium-dependent cell-adhesion |
| SSTR1 | Somatostatin receptor 1 | Regulate diverse cellular functions |
| FOXA2 | Forkhead box A2 | Involved in the development of multiple endoderm-derived organ systems |
| CDO1 | Cysteine dioxygenase, type I | Initiates metabolic pathways，regulate cellular cysteine concentrations. |
| GRIA2 | Glutamate receptor, ionotropic, AMPA2 | Ligand-gated ion channel |
| FRY | Furry homolog (Drosophila) | Maintain structural integrity of mitotic centrosomes |
| SOX17 | SRY (sex determining region Y)-box 17 | Transcriptional regulation |
| HNF1B | HNF1 homeobox B | Transcription factor |
| PHACTR1 | Phosphatase and actin regulator 1 | Cell motility, the formation of tubules |
| TBX5 | T-box 5 | Transcriptional regulation |
| TMC2 | Transmembrane channel-like 2 | Required for the normal function of cochlear hair cells |
| HLF | Hepatic leukemia factor | Activate transcription |
| CLEC14A | C-type lectin domain family 14, member A | Filopodia formation, cell migration and tube formation |
| CA3 | Carbonic anhydrase III, muscle specific | Reversible hydration of carbon dioxide |
| SPTBN1 | Spectrin, beta, non-erythrocytic 1 | Calcium-dependent movement of the cytoskeleton |
| VWF | Von Willebrand factor | Maintenance of hemostasis |
| C7orf41 | Chromosome 7 open reading frame 41 | Undetermined |
| TGFBR3 | Transforming growth factor, beta receptor III | Involved in capturing and retaining TGF-beta for presentation to the signaling receptors |
| ACVRL1 | Activin A receptor type II-like 1 | Regulator of normal blood vessel development |
| TMEM212 | Transmembrane protein 212 | Undetermined |
| TAL1 | T-cell acute lymphocytic leukemia 1 | Implicated in the genesis of hemopoietic malignancies. |
| ARRB1 | Arrestin, beta 1 | Members of arrestin/beta-arrestin protein family |
| COX7A1 | Cytochrome c oxidase subunit VIIa polypeptide 1 | Mitochondrial electron transport |
| PKNOX2 | PBX/knotted 1 homeobox 2 | Cell proliferation, differentiation, and death |
| TBX4 | T-box 4 | Required for mesoderm differentiation. |
| CFTR | Cystic fibrosis transmembrane conductance regulator | Inhibit the chloride channel activity of ANO1. |
| PEAR1 | Platelet endothelial aggregation receptor 1 | Undetermined |
| ADHFE1 | Alcohol dehydrogenase, iron containing, 1 | Undetermined |
| SOD3 | Superoxide dismutase 3, extracellular | Protect the brain, lungs, and other tissues from oxidative stress. |
| PPP1R15A | Protein phosphatase 1, regulatory (inhibitor) subunit 15A | Facilitating recovery of cells from stress，promote apoptosis |
| GRK5 | G protein-coupled receptor kinase 5 | Activate G protein-coupled receptors，regulating the motility of  Polymorphonuclear leukocytes (PMNs). |
| GATA6 | GATA binding protein 6 | Regulating differentiation and/or proliferation |
| CD34 | CD34 molecule | Cell adhesion |
| Top hypomethylated and up-regulated genes in LUSC | | |
| GJB5 | Gap junction protein, beta 5, 31.1kDa | Involved in intercellular communication |
| AKR1B10 | Aldo-keto reductase family 1, member B10 | Acts as all-trans-retinaldehyde reductase. |
| SERPINB5 | Serpin peptidase inhibitor, clade B, member 5 | Tumor suppressor. |
| LOC642587 | NPC-A-5 | Undetermined |
| GJB3 | Gap junction protein, beta 3, 31kDa | Gap junctions |
| TP63 | Tumor protein p63 | Transcriptional activator or repressor. |
| STRA6 | Stimulated by retinoic acid gene 6 homolog (mouse) | Participates in p53-induced apoptosis after DNA damage |
| SLC2A1 | Solute carrier family 2 , member 1 | Glucose transporter |
| RAPGEFL1 | Rap guanine nucleotide exchange factor (GEF)-like 1 | Undetermined |
| CLDN1 | Claudin 1 | Tight junctions |
| GPR87 | G protein-coupled receptor 87 | Necessary for p53/TP53-dependent survival in response to DNA damage |
| DQX1 | DEAQ box RNA-dependent ATPase 1 | Undetermined |
| KRT15 | Keratin 15 | Responsible for the structural integrity of epithelial cells |
| ARNTL2 | Aryl hydrocarbon receptor nuclear translocator-like 2 | Activate E-box element (5'-CACGTG-5') transcription. |
| KRTDAP | Keratinocyte differentiation-associated protein | Regulator of keratinocyte differentiation |
| CENPM | Centromere protein M | Plays a central role in assembly of kinetochore proteins, mitotic progression and chromosome segregation |
| FAM19A4 | Family with sequence similarity 19 (chemokine (C-C motif)-like), member A4 | A member of the TAFA family |
| ZNF750 | Zinc finger protein 750 | Acts downstream of p63/TP63 and activates expression of late epidermal differentiation genes |
| IL-1F9 | Interleukin 1 family, member 9 | Undetermined |
| TRIP13 | Thyroid hormone receptor interactor 13 | Plays a key role in chromosome recombination and chromosome structure development during meiosis. |
| VSNL1 | Visinin-like 1 | Regulates the inhibition of rhodopsin phosphorylation |
| KRT80 | Keratin 80 | Responsible for the structural integrity of epithelial cells |
| TP73 | Tumor protein p73 | Participates in the apoptotic response to DNA damage |
| HMGA2 | High mobility group AT-hook 2 | Cell cycle regulation，chromosome condensation |
| ST18 | Suppression of tumorigenicity 18 | Represses transcription activity |
| IL22RA2 | Interleukin 22 receptor, alpha 2 | Blocks the activity of IL22 |

Supplementary Table 4 Clinicopathological characteristics of patients for TCGA validation cohorts

| Clinical and pathological variables | LUSC  （n=343） | NTL（n=39） |
| --- | --- | --- |
| Age (years) | | |
| < 60 | 61 | 6 |
| ≥ 60 | 282 | 33 |
| Gender | | |
| Male | 255 | 26 |
| Female | 89 | 13 |
| Smoking status | | |
| Current smoker | 104 | 9 |
| Current reformed smoker for ≤15 years | 160 | 18 |
| Current reformed smoker for > 15 years | 54 | 11 |
| Never-smoker | 10 | 1 |
| Clinical stage | | |
| I | 163 |  |
| II | 122 |  |
| III+IV | 57 |  |
| T stage | | |
| T1 | 83 |  |
| T2 | 193 |  |
| T3 | 54 |  |
| T4 | 13 |  |
| N stage | | |
| N0 | 219 |  |
| Nx | 124 |  |
| M stage | | |
| M0 | 264 |  |
| Mx | 79 |  |
| Survival time（years） | | |
| ≤3 | 269 |  |
| 3-5 | 38 |  |
| ≥5 | 36 |  |

Supplementary Table 5 Details of the CGs dinucleotides for these six genes.

| Gene | Ilmn ID | Genbank Accession | Forward Sequence | Gene-based regions | CpG island-based regions |
| --- | --- | --- | --- | --- | --- |
| CLDN1 | cg00804587 | NM_021101 | GGCATAACAGACACATGATATAAGACAATTAATGTGATTTCTCAAGGAAGATACTCCAGC[CG]TGAATCATTATTATCTAAGTAAATGGGAATTTAATGACTTTTCGGACAAGGAACTCGAAA | Body | N_Shore |
| TP63 | cg06520450 | NM_003722 | CATTGGAGTGGAGGAGTCCAGGTGGAAGTTGATGGATTGGACAGGTAAAGAGAAGAGTCC[CG]CCTCCTCATGCCTATAGTTGGGTATATATTAGGAAACCTTAAATTATGTACAGAGAGAGA | Body |  |
| HNF1B | cg19378036 | NM_000458 | GACTTGTTAAGCCAGTGCCTTGCAGCCTAGGCGCGGGGCTTTTCCACCGGTTCTCAGTTC[CG]CTTTAGTCCAGAGAGAAGACACTACGCTTCGGGGTTCAGGTGAGAGCCAGAGGAAGCTGG | TSS1500 | Island |
| TBX5 | cg23827572 | NM_080718 | CTTATTATTATTATTATTATTTTTAAATCCCCCGCGGAGGAGCTCTGGGCAAATGAATAC[CG]AGGCGCCGCTCTAGCTGGTTAGGCTTGGGATGCGATAACTCAGTGCCCTCTTGCAGACTT | TSS200 | Island |
| TCF21 | cg04692403 | NM_003206 | AGCCAGGAGGGGAAGCAGGTCCAGCGCAACGCCGCCAACGCGCGAGAGCGGGCCCGCATG[CG]AGTGCTGAGCAAGGCCTTCTCCAGACTCAAGACCACCCTGCCCTGGGTGCCCCCCGACAC | 1st Exon | Island |
| ADHFE1 | cg09383816 | NM_144650 | CCCGAGGCCTACGGAGCAGTTACCTTCTACGGCAATTTCAAGGGTGGATGGTGCGAGCGC[CG]CTGGGGCAGCTGGCGTTCTGGTTCTTACTCCGTGGGAAAATGGCCCTGAGCCCGACTGGC | TSS200 | Island |
